# Supplementary material for: A Likelihood Approach for Real-Time Calibration of Stochastic Compartmental Epidemic Models
Source: PLoS Comput Biol. 2017 Jan 17;13(1):e1005257. doi: 10.1371/journal.pcbi.1005257 (PMC5240920; doi:10.1371/journal.pcbi.1005257)
Supplement: S1 File — (TAR.GZ) [file pcbi.1005257.s014.tar.gz › HSPH_Online-SI-Revision/IPoi09/n10-mild/n10-mild_BENCH2_table-4.pdf]

1	newly 3 weeks spec	199.	411.3	{118., 543.}	122.496	0.37144	newly 3 weeks cumul	583.	1227.9	{267., 1659.}	130.023	0.393761	newly 5 weeks spec	174.	391.8	{217., 550.}	130.307	0.356574	newly 5 weeks cumul	973.	2037.	{658., 2582.}	121.816	0.357786	newly 54 weeks cumul	2959.	4590.53	{2878., 5905.}	57.0666	0.185267
2	newly 3 weeks spec	442.	751.	{584., 946.}	70.2357	0.223426	newly 3 weeks cumul	1121.	2001.88	{1395., 2720.}	80.6049	0.245566	newly 5 weeks spec	488.	721.349	{416., 986.}	56.0522	0.189435	newly 5 weeks cumul	2106.	3488.28	{2916., 4232.}	65.6353	0.214481	newly 59 weeks cumul	3790.	5713.74	{4245., 7064.}	52.2114	0.176539
3	newly 3 weeks spec	438.	102.952	{21., 189.}	76.4949	0.768759	newly 3 weeks cumul	1008.	719.667	{259., 1247.}	49.7402	0.327059	newly 5 weeks spec	488.	13.9524	{0., 39.}	97.1409	2.30504	newly 5 weeks cumul	1997.	766.143	{262., 1359.}	64.1009	0.543034	newly 62 weeks cumul	4433.	778.952	{262., 1379.}	82.4283	0.873715
4	newly 3 weeks spec	427.	518.974	{368., 681.}	30.2108	0.119401	newly 3 weeks cumul	1020.	1678.21	{1039., 2091.}	67.5867	0.217837	newly 5 weeks spec	527.	362.128	{196., 559.}	35.6542	0.21975	newly 5 weeks cumul	2089.	2482.56	{1977., 3140.}	25.2691	0.100726	newly 57 weeks cumul	4089.	3425.85	{2405., 4752.}	21.449	0.115979
5	newly 3 weeks spec	105.	210.407	{109., 276.}	104.762	0.30449	newly 3 weeks cumul	340.	650.185	{396., 828.}	95.2614	0.286063	newly 5 weeks spec	120.	184.333	{88., 264.}	60.7099	0.198252	newly 5 weeks cumul	593.	1027.48	{599., 1355.}	77.2656	0.242814	newly 37 weeks cumul	2494.	2851.	{1490., 5051.}	38.9008	0.153302
6	newly 3 weeks spec	329.	584.923	{242., 989.}	93.2897	0.39845	newly 3 weeks cumul	816.	2208.65	{1388., 2887.}	184.771	0.602816	newly 5 weeks spec	353.	227.	{84., 458.}	52.3861	0.446502	newly 5 weeks cumul	1522.	2805.65	{1566., 3985.}	96.6567	0.438652	newly 63 weeks cumul	3219.	3232.54	{2224., 4767.}	27.9279	0.276579
7	newly 3 weeks spec	337.	497.962	{257., 741.}	56.3684	0.183178	newly 3 weeks cumul	914.	1739.96	{1274., 2247.}	90.3678	0.269184	newly 5 weeks spec	288.	269.731	{86., 506.}	47.289	0.242215	newly 5 weeks cumul	1547.	2384.77	{1612., 3246.}	55.7605	0.180787	newly 59 weeks cumul	3151.	2855.31	{1799., 4140.}	25.4498	0.126089
8	newly 3 weeks spec	276.	477.933	{262., 625.}	77.4879	0.242625	newly 3 weeks cumul	750.	1485.	{1025., 1789.}	101.289	0.300451	newly 5 weeks spec	242.	385.333	{166., 567.}	68.9256	0.216647	newly 5 weeks cumul	1290.	2309.3	{1413., 2835.}	80.1835	0.246331	newly 54 weeks cumul	4074.	3829.33	{1902., 5290.}	25.9221	0.126054
9	newly 3 weeks spec	372.	494.387	{273., 721.}	43.8952	0.158162	newly 3 weeks cumul	848.	1622.55	{1069., 1970.}	91.6274	0.269751	newly 5 weeks spec	527.	328.742	{139., 550.}	41.6845	0.2877	newly 5 weeks cumul	1906.	2366.19	{1601., 3123.}	31.7673	0.119457	newly 58 weeks cumul	4128.	3245.26	{1854., 4444.}	25.354	0.143865
10	newly 3 weeks spec	221.	166.5	{36., 309.}	64.6189	0.395151	newly 3 weeks cumul	630.	696.154	{231., 1307.}	55.2991	0.239518	newly 5 weeks spec	246.	75.9615	{14., 128.}	76.7824	0.741139	newly 5 weeks cumul	1140.	891.769	{265., 1688.}	54.4872	0.316505	newly 55 weeks cumul	2746.	1012.08	{282., 1789.}	68.4856	0.581763
11	newly 3 weeks spec	268.	362.941	{119., 599.}	60.2283	0.218073	newly 3 weeks cumul	729.	1373.29	{712., 1947.}	90.2041	0.25369	newly 5 weeks spec	221.	177.588	{43., 377.}	58.2912	0.349474	newly 5 weeks cumul	1225.	1808.53	{786., 2772.}	60.0144	0.19668	newly 60 weeks cumul	2530.	2055.41	{816., 3256.}	34.9314	0.196387
12	newly 3 weeks spec	699.	670.373	{271., 1007.}	34.1552	0.222374	newly 3 weeks cumul	1437.	1468.	{469., 2557.}	45.0325	0.264811	newly 5 weeks spec	522.	868.647	{454., 1173.}	77.7665	0.261472	newly 5 weeks cumul	2666.	3154.18	{1486., 4407.}	40.249	0.198001	newly 62 weeks cumul	3704.	6896.14	{4957., 8753.}	86.1808	0.2616
13	newly 3 weeks spec	431.	476.983	{147., 677.}	42.0611	0.214774	newly 3 weeks cumul	1134.	1238.37	{339., 1915.}	47.9012	0.247403	newly 5 weeks spec	505.	551.917	{321., 694.}	26.0627	0.135671	newly 5 weeks cumul	2205.	2312.3	{857., 3181.}	35.8534	0.192366	newly 54 weeks cumul	4115.	7228.58	{5816., 8156.}	75.6642	0.241649
14	newly 3 weeks spec	356.	632.32	{480., 758.}	79.5618	0.249763	newly 3 weeks cumul	939.	1818.78	{1180., 2213.}	96.5602	0.286342	newly 5 weeks spec	351.	563.88	{397., 688.}	61.7778	0.201538	newly 5 weeks cumul	1659.	3007.14	{2451., 3443.}	82.2652	0.256232	newly 55 weeks cumul	3922.	5550.34	{4202., 6842.}	42.6767	0.148933
15	newly 3 weeks spec	198.	264.2	{90., 492.}	64.6898	0.240247	newly 3 weeks cumul	512.	920.286	{394., 1547.}	91.6406	0.281233	newly 5 weeks spec	238.	177.114	{40., 385.}	56.1945	0.370643	newly 5 weeks cumul	1009.	1316.11	{524., 2227.}	57.6044	0.218186	newly 57 weeks cumul	4011.	2003.03	{794., 4110.}	51.2227	0.390569
16	newly 3 weeks spec	383.	565.778	{362., 732.}	50.1402	0.168255	newly 3 weeks cumul	938.	1748.63	{1115., 2268.}	87.4319	0.263283	newly 5 weeks spec	435.	407.407	{212., 593.}	28.4802	0.137213	newly 5 weeks cumul	1811.	2651.3	{1944., 3252.}	46.69	0.16011	newly 58 weeks cumul	3950.	3715.11	{2479., 4633.}	17.0445	0.0821759
17	newly 3 weeks spec	66.	136.826	{10., 296.}	133.992	0.392078	newly 3 weeks cumul	166.	335.	{28., 721.}	130.801	0.390265	newly 5 weeks spec	111.	200.13	{28., 365.}	105.131	0.378843	newly 5 weeks cumul	390.	703.609	{62., 1452.}	108.919	0.367907	newly 55 weeks cumul	3319.	6455.52	{5216., 7374.}	94.502	0.285456
18	newly 3 weeks spec	196.	220.091	{24., 416.}	64.4712	0.362023	newly 3 weeks cumul	553.	769.909	{147., 1269.}	68.2394	0.294007	newly 5 weeks spec	192.	154.227	{19., 335.}	71.2831	0.481704	newly 5 weeks cumul	970.	1113.36	{235., 1946.}	58.7816	0.299397	newly 55 weeks cumul	2464.	1603.82	{312., 3417.}	53.7043	0.419728
19	newly 3 weeks spec	378.	408.533	{154., 741.}	47.1605	0.205712	newly 3 weeks cumul	1189.	1637.93	{859., 2353.}	50.3056	0.173778	newly 5 weeks spec	370.	188.833	{48., 328.}	61.9009	0.466203	newly 5 weeks cumul	1992.	2107.77	{975., 3378.}	36.6951	0.161111	newly 58 weeks cumul	3457.	2419.43	{1054., 3775.}	39.4147	0.240172
20	newly 3 weeks spec	289.	671.536	{345., 938.}	132.365	0.342989	newly 3 weeks cumul	900.	2267.36	{1710., 2802.}	151.929	0.394525	newly 5 weeks spec	356.	372.357	{134., 756.}	46.0674	0.205239	newly 5 weeks cumul	1637.	3162.89	{2056., 4082.}	93.2128	0.274453	newly 59 weeks cumul	3969.	3825.46	{2299., 5356.}	22.9196	0.107878
21	newly 3 weeks spec	420.	836.2	{428., 1018.}	103.278	0.303931	newly 3 weeks cumul	1015.	2198.	{893., 2912.}	123.934	0.342951	newly 5 weeks spec	456.	797.517	{484., 1006.}	75.1864	0.231664	newly 5 weeks cumul	1966.	3845.5	{2455., 4506.}	97.5348	0.290594	newly 59 weeks cumul	3646.	6699.93	{5305., 7639.}	83.7612	0.260559
22	newly 3 weeks spec	279.	573.754	{355., 736.}	112.592	0.327936	newly 3 weeks cumul	788.	1474.61	{783., 2070.}	96.0119	0.292445	newly 5 weeks spec	256.	640.262	{519., 754.}	150.961	0.391332	newly 5 weeks cumul	1343.	2742.74	{1890., 3408.}	109.679	0.319586	newly 55 weeks cumul	3250.	6785.33	{5566., 7751.}	108.779	0.315919
23	newly 3 weeks spec	399.	795.958	{355., 1083.}	108.864	0.320991	newly 3 weeks cumul	1073.	1834.18	{651., 2855.}	85.7462	0.278449	newly 5 weeks spec	394.	973.169	{778., 1107.}	147.97	0.38821	newly 5 weeks cumul	1876.	3726.3	{2047., 4824.}	104.234	0.305541	newly 60 weeks cumul	3098.	8550.23	{7767., 9188.}	175.992	0.439916
24	newly 3 weeks spec	199.	597.86	{374., 789.}	201.618	0.458966	newly 3 weeks cumul	621.	1849.58	{1191., 2423.}	201.253	0.468507	newly 5 weeks spec	188.	474.32	{217., 736.}	154.34	0.366282	newly 5 weeks cumul	1038.	2870.16	{1955., 3666.}	177.118	0.427861	newly 56 weeks cumul	2085.	4632.5	{2592., 6460.}	122.495	0.32273
25	newly 3 weeks spec	253.	347.222	{74., 650.}	78.8538	0.323767	newly 3 weeks cumul	550.	651.389	{131., 1267.}	72.2525	0.340142	newly 5 weeks spec	366.	758.194	{254., 1121.}	121.843	0.369209	newly 5 weeks cumul	1317.	1948.76	{532., 3474.}	76.6525	0.294912	newly 63 weeks cumul	2982.	8864.25	{8263., 9349.}	197.259	0.472224
26	newly 3 weeks spec	272.	204.73	{71., 458.}	47.188	0.27697	newly 3 weeks cumul	780.	831.919	{382., 1598.}	40.8281	0.181786	newly 5 weeks spec	259.	112.568	{28., 330.}	68.225	0.542219	newly 5 weeks cumul	1322.	1098.49	{444., 2357.}	41.023	0.225563	newly 54 weeks cumul	2407.	1358.03	{509., 3230.}	54.1776	0.370122
27	newly 3 weeks spec	426.	562.	{249., 755.}	49.4131	0.218058	newly 3 weeks cumul	1154.	1473.55	{494., 2136.}	51.4948	0.237862	newly 5 weeks spec	412.	597.983	{407., 779.}	52.1723	0.204775	newly 5 weeks cumul	2041.	2668.48	{1408., 3413.}	45.6974	0.204177	newly 56 weeks cumul	3536.	6144.67	{4525., 7087.}	73.7745	0.234393
28	newly 3 weeks spec	120.	163.032	{80., 281.}	51.2903	0.171986	newly 3 weeks cumul	339.	526.097	{317., 842.}	61.1666	0.1885	newly 5 weeks spec	111.	141.323	{63., 247.}	51.3223	0.187086	newly 5 weeks cumul	576.	815.323	{482., 1373.}	51.9321	0.166763	newly 34 weeks cumul	2091.	2010.	{922., 3709.}	47.7114	0.222363
29	newly 3 weeks spec	361.	716.373	{504., 903.}	99.3754	0.289592	newly 3 weeks cumul	1007.	2064.08	{1288., 2655.}	107.395	0.304461	newly 5 weeks spec	360.	639.49	{362., 873.}	81.8954	0.248644	newly 5 weeks cumul	1758.	3396.43	{2526., 4140.}	93.5444	0.279225	newly 57 weeks cumul	3678.	5680.39	{4146., 6946.}	57.0909	0.191156
30	newly 3 weeks spec	367.	506.469	{157., 774.}	62.3367	0.238877	newly 3 weeks cumul	970.	1707.27	{717., 2296.}	85.2135	0.26408	newly 5 weeks spec	361.	344.51	{62., 729.}	53.8866	0.305797	newly 5 weeks cumul	1701.	2479.8	{878., 3599.}	63.8052	0.229131	newly 56 weeks cumul	3445.	3464.45	{1108., 5893.}	39.8857	0.203944
31	newly 3 weeks spec	288.	677.097	{376., 964.}	148.006	0.581345	newly 3 weeks cumul	670.	1669.61	{774., 2423.}	162.234	0.588655	newly 5 weeks spec	360.	617.742	{182., 894.}	91.0573	0.483438	newly 5 weeks cumul	1418.	2985.29	{2119., 3933.}	123.427	0.556702	newly 63 weeks cumul	2824.	4316.16	{2805., 5478.}	66.4135	0.449185
32	newly 3 weeks spec	126.	206.395	{112., 307.}	70.3027	0.209897	newly 3 weeks cumul	395.	665.372	{404., 993.}	71.7221	0.215579	newly 5 weeks spec	145.	172.	{80., 279.}	47.6504	0.188665	newly 5 weeks cumul	687.	1025.07	{571., 1522.}	58.1869	0.185314	newly 36 weeks cumul	3008.	2339.95	{1057., 3917.}	43.2088	0.245121
33	newly 3 weeks spec	466.	629.183	{357., 853.}	50.136	0.195702	newly 3 weeks cumul	1168.	1553.27	{687., 2320.}	54.7921	0.216498	newly 5 weeks spec	533.	737.408	{606., 857.}	44.2909	0.165496	newly 5 weeks cumul	2236.	2987.11	{1774., 3965.}	45.9882	0.178216	newly 57 weeks cumul	4145.	7722.11	{6554., 8564.}	86.2995	0.268072
34	newly 3 weeks spec	301.	449.065	{317., 593.}	53.5848	0.17815	newly 3 weeks cumul	838.	1555.9	{1125., 1969.}	86.5232	0.260936	newly 5 weeks spec	335.	275.903	{154., 421.}	34.3187	0.184645	newly 5 weeks cumul	1525.	2199.23	{1646., 2744.}	48.5732	0.168465	newly 57 weeks cumul	3835.	2892.19	{1961., 4045.}	28.9549	0.163222
35	newly 3 weeks spec	430.	736.816	{481., 958.}	71.3526	0.223105	newly 3 weeks cumul	1171.	2346.24	{1863., 2715.}	100.363	0.296998	newly 5 weeks spec	358.	505.878	{256., 826.}	52.4684	0.173451	newly 5 weeks cumul	1960.	3481.47	{2514., 4191.}	77.626	0.243213	newly 57 weeks cumul	3634.	4896.18	{3146., 6557.}	39.3612	0.137743
36	newly 3 weeks spec	452.	543.441	{246., 879.}	45.7509	0.189385	newly 3 weeks cumul	1197.	1896.38	{1161., 2357.}	64.4184	0.210003	newly 5 weeks spec	398.	294.912	{87., 526.}	49.1502	0.316282	newly 5 weeks cumul	2080.	2608.35	{1421., 3863.}	40.7579	0.15806	newly 59 weeks cumul	3700.	3136.09	{1700., 4774.}	30.0914	0.167086
37	newly 3 weeks spec	333.	680.071	{336., 911.}	110.618	0.321634	newly 3 weeks cumul	782.	2097.5	{1477., 2805.}	174.452	0.450928	newly 5 weeks spec	357.	412.214	{105., 932.}	53.3413	0.230969	newly 5 weeks cumul	1618.	3059.46	{2102., 3930.}	94.0645	0.289882	newly 62 weeks cumul	2716.	3918.11	{2258., 5783.}	49.4333	0.167262
38	newly 3 weeks spec	369.	522.185	{264., 754.}	53.1567	0.181651	newly 3 weeks cumul	949.	1718.04	{1184., 2245.}	82.223	0.248761	newly 5 weeks spec	348.	339.519	{108., 600.}	43.1992	0.216857	newly 5 weeks cumul	1684.	2490.48	{1480., 3392.}	52.5534	0.175113	newly 58 weeks cumul	4183.	3276.89	{1686., 4831.}	28.1395	0.15765
39	newly 3 weeks spec	526.	364.294	{110., 631.}	38.3695	0.254602	newly 3 weeks cumul	1168.	1296.12	{484., 1913.}	35.2337	0.165826	newly 5 weeks spec	460.	211.529	{83., 472.}	54.6803	0.444651	newly 5 weeks cumul	2156.	1783.47	{590., 2571.}	29.8237	0.171596	newly 59 weeks cumul	3091.	2383.29	{1341., 3760.}	30.6259	0.185476
40	newly 3 weeks spec	95.	204.167	{150., 275.}	117.579	0.329545	newly 3 weeks cumul	322.	631.3	{412., 764.}	99.7205	0.298238	newly 5 weeks spec	79.	184.967	{137., 262.}	134.135	0.355703	newly 5 weeks cumul	527.	1009.37	{750., 1188.}	94.1872	0.283276	newly 32 weeks cumul	1882.	2905.73	{1592., 4760.}	60.4676	0.183985
41	newly 3 weeks spec	565.	390.273	{147., 708.}	46.4039	0.309056	newly 3 weeks cumul	1036.	1609.95	{655., 2464.}	67.9493	0.222587	newly 5 weeks spec	573.	113.227	{21., 206.}	80.2396	0.856145	newly 5 weeks cumul	2224.	1954.36	{1132., 2919.}	31.6138	0.169455	newly 63 weeks cumul	3132.	2055.86	{1221., 3071.}	36.7511	0.23477
42	newly 3 weeks spec	232.	297.364	{137., 535.}	50.627	0.183504	newly 3 weeks cumul	680.	1019.	{566., 1426.}	56.484	0.187084	newly 5 weeks spec	278.	207.545	{66., 491.}	60.6279	0.37229	newly 5 weeks cumul	1255.	1477.55	{708., 2312.}	39.2177	0.154181	newly 55 weeks cumul	2911.	2087.64	{829., 3815.}	42.4065	0.257575
43	newly 3 weeks spec	534.	489.235	{190., 747.}	25.9639	0.165968	newly 3 weeks cumul	1357.	1526.53	{392., 2155.}	36.5425	0.207261	newly 5 weeks spec	396.	364.294	{110., 631.}	41.1319	0.218884	newly 5 weeks cumul	2311.	2333.41	{874., 3187.}	24.4228	0.144482	newly 61 weeks cumul	3391.	3420.59	{2712., 4190.}	16.5299	0.0753383
44	newly 3 weeks spec	300.	616.078	{344., 819.}	111.059	0.31362	newly 3 weeks cumul	794.	1672.39	{702., 2336.}	120.275	0.332143	newly 5 weeks spec	309.	613.51	{383., 800.}	101.504	0.292394	newly 5 weeks cumul	1422.	2915.18	{1739., 3664.}	109.211	0.31087	newly 57 weeks cumul	3738.	5914.88	{4389., 7090.}	60.3505	0.200398
45	newly 3 weeks spec	534.	806.75	{179., 1277.}	77.1139	0.286428	newly 3 weeks cumul	994.	1613.35	{334., 2873.}	91.4448	0.319122	newly 5 weeks spec	717.	1109.87	{545., 1527.}	65.4517	0.219378	newly 5 weeks cumul	2450.	3772.88	{1398., 5474.}	68.9702	0.240856	newly 64 weeks cumul	4278.	7263.37	{5261., 9200.}	70.4422	0.221969
46	newly 3 weeks spec	331.	477.647	{224., 704.}	56.3533	0.187073	newly 3 weeks cumul	825.	1569.41	{1059., 2132.}	90.2317	0.263621	newly 5 weeks spec	370.	257.647	{73., 466.}	46.0095	0.298027	newly 5 weeks cumul	1578.	2190.	{1251., 2950.}	45.0235	0.153411	newly 61 weeks cumul	3207.	2614.47	{1356., 3705.}	27.9591	0.153002
47	newly 3 weeks spec	416.	480.351	{75., 798.}	55.4249	0.492716	newly 3 weeks cumul	828.	1166.84	{158., 2156.}	81.1431	0.519013	newly 5 weeks spec	448.	556.946	{163., 835.}	53.0345	0.459965	newly 5 weeks cumul	1770.	2257.59	{445., 3557.}	58.838	0.477018	newly 61 weeks cumul	4274.	5001.51	{2992., 7514.}	37.9188	0.4227
48	newly 3 weeks spec	360.	571.456	{321., 802.}	64.7612	0.206758	newly 3 weeks cumul	945.	1374.18	{662., 2262.}	59.0606	0.19604	newly 5 weeks spec	310.	736.561	{604., 860.}	137.6	0.369861	newly 5 weeks cumul	1682.	2781.16	{1780., 3770.}	68.0581	0.215004	newly 58 weeks cumul	3197.	7240.21	{6255., 8043.}	126.469	0.35174
49	newly 3 weeks spec	197.	179.273	{57., 418.}	59.8062	0.335251	newly 3 weeks cumul	473.	673.409	{311., 1328.}	71.5837	0.244017	newly 5 weeks spec	183.	102.182	{32., 301.}	72.3795	0.590191	newly 5 weeks cumul	887.	911.318	{389., 2158.}	56.4979	0.267041	newly 54 weeks cumul	3344.	1158.18	{412., 3201.}	65.5013	0.591158
50	newly 3 weeks spec	233.	133.857	{50., 209.}	60.8216	0.437375	newly 3 weeks cumul	705.	566.952	{271., 998.}	47.3556	0.280703	newly 5 weeks spec	204.	65.2857	{9., 133.}	74.9066	0.770345	newly 5 weeks cumul	1157.	723.714	{345., 1306.}	54.7598	0.374877	newly 54 weeks cumul	2093.	837.619	{355., 1492.}	66.4141	0.550292
